# Clinical and psychosocial changes in adults with opioid use disorder and chronic pain using medical cannabis: a brief report

**DOI:** 10.1186/s42238-025-00297-5

**Published:** 2025-06-18

**Authors:** Michelle R. Lent, Ryan Keen, Michael Ruiz, Hannah R. Callahan, Katherine E. Galluzzi, Karen L. Dugosh

**Affiliations:** 1 Philadelphia College of Osteopathic Medicine, Department of Clinical Psychology, 4170 City Avenue, Rowland Hall, Philadelphia, PA 19131 USA; 2 Philadelphia College of Osteopathic Medicine, Department of Geriatric & Palliative Medicine, Philadelphia, PA USA; 3 Public Health Management Corporation, Research & Evaluation Group, Philadelphia, PA USA

**Keywords:** Cannabis, Opioid use disorder, Chronic pain

## Abstract

**Background:**

Medical cannabis (MC) is approved for the treatment of opioid use disorder (OUD) in Pennsylvania, but little is known about how MC impacts illicit opioid use or the clinical and psychosocial factors including pain severity levels that can precede illicit opioid use. This observational study examined the extent to which changes in rates of illicit opioid use and in pain and psychosocial functioning were observed following the first three months of MC treatment.

**Methods:**

A referred sample of 47 adults taking buprenorphine/naloxone for OUD with a minimum pain severity rating of 5/10 enrolled from March 2022–April 2023. Participants were recruited from an outpatient MC physician recommender’s office and were offered a discounted MC 1:1 tetrahydrocannabinol:cannabidiol 5 mg:5 mg daily oral capsule. The primary study outcomes were pain severity, self-efficacy and interference, and the rates of illicit substance use as assessed via urine drug screening (UDS).

**Results:**

Participants (64% male, 49% Black, average age = 44 years) reported significant decreases in pain severity from baseline (*M* = 5.18, *SD* = 2.09) to Month 3 (*M* = 4.39, *SD* = 2.28), *P* < 0.01, Cohen’s *d* = 0.54, and pain interference from baseline (*M* = 5.21, *SD* = 2.79) to Month 3 (*M* = 4.32, *SD* = 2.86), *P* < 0.01, Cohen’s *d* = 0.47, and increases in pain-related self-efficacy from baseline (*M* = 6.55, *SD* = 3.57) to Month 3 (*M* = 8.05, *SD* = 3.30), *P* < 0.01, Cohen’s *d* = 0.44. Rates of opioid use (*X*^2^[1] = 4.00, *P* = 0.13) did not differ significantly from baseline (16%) to Month 3 (5%). Cravings for opioids were mildly higher at baseline (*M* = 2.15, *SD* = 2.88) than at 3-months (*M* = 1.78, *SD* = 2.95) but this difference was not statistically significant, *P* = 0.49, *d* = 0.1. Sleep quality scores improved significantly from baseline (*M* = 12.38, *SD* = 4.40) to Month 3 (*M* = 10.95, *SD* = 4.95), *P* < 0.05, *d* = 0.33. Quality of life significantly improved in seven of eight domains (*P* < 0.05).

**Conclusion:**

MC treatment initiation was associated with reductions in pain severity and interference and improvements in quality of life and sleep quality, but not in illicit opioid use or cravings in adults with chronic pain receiving buprenorphine/naloxone for OUD.

## Background

Recent legislation, such as the Medication Access and Training Expansion (MATE) Act signed in 2022, has enabled greater access to effective pharmacologic treatment for opioid use disorder (OUD) (Hughes et al. 2023 Oct 1). However, many individuals with OUD continue to face challenges with the management of chronic pain, which may relate to limits on addiction treatment providers’ resources or capacity (Ellis et al. 2021). An estimated 34–64% of individuals with OUD live with comorbid pain conditions (Ellis et al. 2021; Hser et al. 2017; Baumann and Samuels 2022); moreover, 66% report challenges with pain management and 47% report that their pain is actually worsening (Ellis et al. 2021). When not sufficiently addressed, chronic pain can be associated with adverse outcomes in individuals with substance use disorders including negative mood, cravings to use substances, and diminished quality of life (Ellis et al. 2021; Ferguson et al. 2022; Barry et al. 2008; Stack et al. 2000). Although certain opioid agonist and partial agonist medications for OUD (MOUDs) such as buprenorphine/naloxone are associated with positive substance use-related outcomes and are FDA-approved for pain management (Dalal et al. 2021; Malinoff et al. 2005), a substantial proportion of patients may continue to experience pain (Dalal et al. 2021; Malinoff et al. 2005). Little is known regarding the use of alternative pain management strategies such as medical cannabis (MC) for individuals using MOUD. Several studies including randomized controlled trials found cannabis to be associated with analgesic effects in a variety of pain-related conditions (Hill 2015; Whiting et al. 2015; Wallace et al. 2015). Moreover, evidence suggests the potential for a synergistic effect of cannabis and opioids in regards to pain management given that when cannabinoids are co-administered with opioids, reduced opioid doses may be equally efficacious (Nielsen et al. 2017; Foll 2021). Cannabis may also positively attenuate cravings to use opioids in OUD (Reddon et al. 2023; Hurd et al. 2019), improve sleep (AminiLari et al. 2022), and help with withdrawal symptoms (Wiese and Wilson-Poe 2018), which may serve as potential antecedents to substance use (Ferguson et al. 2021; Kleykamp et al. 2019; Kosten and Baxter 2019; Ara et al. 2016).

The availability of high-quality evidence supporting the use of cannabis to aid in OUD recovery remains limited, primarily due to the Schedule I status of cannabis (Suzuki and Weiss 2021). Currently, MC is approved in Pennsylvania (PA) for the treatment of chronic pain (severe/intractable) and OUD (in combination with primary therapeutic interventions) with the recommendation of a physician.

The objectives of this observational intervention study were to examine the extent to which changes in the rates of illicit substance use and pain-related outcomes are observed over the first three months following MC treatment initiation in individuals using MOUD (buprenorphine) and experiencing chronic pain. We hypothesized that participants would report significant improvements in pain symptoms and have significantly lower rates of substance use from baseline (prior to MC use) to three months following MC treatment initiation. Secondary outcomes examined included sleep quality, quality of life, and cravings as they represent potential mechanisms of actions for any observed changes in pain functioning and opioid use.

## Methods

Individuals aged 18 years + were eligible if they had a recommendation from a certified PA physician for MC for OUD but had not yet started using MC, were taking extended-release buprenorphine or buprenorphine/naloxone for OUD (sublingual or injectable), and reported a minimum pain score of 5 (out of 10) on the following question of the Brief Pain Inventory–Short Form: “Please rate your pain by marking the box beside the number that best describes your pain on the average"(Cleeland and Ryan 1994). Individuals were screened for eligibility using the following definition of chronic pain: “History of chronic pain, as documented in the medical record or at baseline assessment, including persistent pain lasting 12 weeks or longer and functional impairment or distress related to pain.” Individuals were not excluded if they reported prior recreational cannabis use, but were excluded if they could not conduct consent in English, if cannabis was prohibited at their residence, or because of criminal justice system involvement that considered cannabis use a violation. At the writing of this report, cannabis was only permitted for medical use and not permitted for sale for recreational use in PA. Potentially eligible individuals were introduced to research staff at the outpatient office of a certified MC physician recommender following their initial MC consultation from March 2022–April 2023. Interested individuals completed the informed consent process that included allowing access to their dispensary and medical records, completed surveys via a structured interview, and provided a urine sample for substance use testing. The research team did not provide advice or guidance regarding the use of recreational cannabis or other substances. It is possible that guidance in this regard was provided by their recommending physician. No prospective assignment to MC occurred as participants had already obtained a MC recommendation prior to consent.

Participants were offered a discount at a MC dispensary on one MC product for the duration of participation, which was a 1:1 tetrahydrocannabinol:cannabidiol (THC:CBD) 5 mg:5 mg oral capsule (30-day supply for $1 USD). The formulation aligns with recommendations for cannabis use for chronic pain (MacCallum et al. 2021; Busse et al. 2021; Seehusen and Kehoe 2022). Participants were permitted to purchase any MC product in addition to the capsules. Participants’ MC purchases from all PA dispensaries were collected using the research version of the state tracking software, MJ Freeway. Participants were remunerated $30 USD per study visit.

Participants were followed for one year and the present study analyzed interim data from baseline to Month 3 post-enrollment. Data were managed using REDCap (Research Electronic Data Capture) (Harris et al. 2009). The Philadelphia College of Osteopathic Medicine’s Institutional Review Board (#H18-054) approved the protocol and provided ethical oversight. Severe adverse events (SAEs) were monitored and defined as death or illness/injuries requiring hospitalization, or that resulted in permanent damage.

### Measures

Study outcomes were collected at baseline and/or Month 3 post-enrollment and included:

- *Demographic and Medical Information.* Participants reported their age, gender, race/ethnicity, employment, body mass index, diagnoses, and medications. The Prescription Drug Monitoring Program (PDMP) was utilized to determine buprenorphine utilization.
- *Substance Use.* Participants provided a urine sample for substance use testing using a 14-panel cup, which was also tested for fentanyl with a test strip (UDS; 14-Panel Drug Test Cup with EDDP/6 Adulterants-Identify Diagnostics, USA CLIA Waived). A UDS was coded positive for opioids if any of the following panels/strips were positive: fentanyl, unexcused methadone, oxycodone, opiate (heroin, morphine, codeine). Buprenorphine testing is included on the panel but not in the aforementioned opioid use definition. Current (past 90 days) recreational cannabis use was measured using a modified question from the Addiction Severity Index-5 drug grid (“Recreational Cannabis”) (McLellan et al. 1992).
- *Pain.* Participants completed the Pain Self-Efficacy Questionnaire Short Form (PSEQ-2) (Nicholas et al. 2015), a questionnaire assessing functional status in relation to pain, and the Brief Pain Inventory-Short Form (BPI), an assessment of the severity of pain and its impact on functioning (Cleeland and Ryan 1994). PSEQ-2 scores range from 0–12 with higher scores indicating greater self-efficacy to manage pain, and BPI severity and intensity subscale scores can range from 1–10 with higher scores indicating greater severity and intensity.
- *Health-Related Quality of Life (HRQoL).* Participants completed the Short Form-36 (SF-36), an assessment evaluating eight domains of physical and mental health functioning over the past four weeks (Ware and Sherbourne 1992; Hays et al. 1993). Scores for each domain range from 0–100 with higher scores indicating better functioning.
- *Sleep​.* Participants completed the Pittsburgh Sleep Quality Index (PSQI) (Buysse et al. 1989), a 19-item assessment of sleep quality over onemonthl. Global scores can range from 0–21 with higher scores indicating worse sleep quality.
- *Craving​s.* Participants completed the Brief Substance Craving Scale (BSCS) (Somoza et al. 1995), a measure of drug craving intensity, duration, and frequency over 24 h. Craving scores can range from 0–12 with higher scores indicating higher levels of craving.
- *MC Purchases.* Product name, type, and percentage THC/CBD were collected from the seed-to-sale PA cannabis product tracking software, MJ Freeway, for all participant MC purchases from baseline through Month 3. MJ Freeway contains MC purchase information from all PA dispensaries.

### Statistical analyses

Descriptive statistics were computed to characterize the sample at baseline and to describe MC purchases. Dependent t-tests and McNemar tests were used to examine changes in each outcome variable from baseline to Month 3. Effect sizes (Cohen’s *d* and odds ratios) were calculated to inform the extent to which observed changes in outcomes were clinically meaningful. Significance levels were set at *P* < 0.05.

## Results

### Participants

Of the 58 individuals approached, two were excluded for not meeting eligibility criteria and nine declined citing time burden. Among the 47 individuals who enrolled, 40 (85%) completed the 3-month follow-up assessment battery and 38 (81%) completed the UDS. Two SAEs (inpatient alcohol detox, psychiatric hospitalization) were reported.

Participant demographics are presented for the overall sample (*N* = 47) in Table 1. Overall, participants were not buprenorphine-naïve. Based on PDMP data for the year prior to study enrollment (*n* = 45), only seven had first listed buprenorphine prescriptions in the 180 days prior to study entry, with four having first listed buprenorphine prescriptions in the 30 days prior to study entry. Within the follow-up sample (*n* = 38), 84% provided a buprenorphine positive UDS at baseline compared with 82% at Month 3 (*P* = 0.65). Females were more likely to complete the Month 3 assessment than males (*X*^*2(*^Hughes et al. 2023 Oct 1) = 4.66, *P* < 0.05); no other baseline differences were observed between those who did and did not complete the Month 3 assessment.

**Table 1:** Sample characteristics and changes in outcomes from baseline to Month 3 in new medical cannabis users with opioid use disorder and chronic pain (*N* = 47)

			*Baseline (N* = *47)*						
Variable			*M/n (range)*	*SD/%*					
Age			44.73 (22.00–63.00)	9.61					
Gender		Male	30	64%					
		Female	17	36%					
Race		White	18	38%					
		Black	23	49%					
		Other	6	13%					
Hispanic/Latino			6	13%					
Employment		Full time	10	21%					
		Part time	8	17%					
		Unemployed	24	51%					
		Disability	5	11%					
Body mass index (kg/m^2^)			29.01 (18.55–48.11)	7.11					
Other medications			31	66%					
		Non-opioid analgesic	17	36%					
		Anxiolytic	12	26%					
		Antidepressant	13	28%					
Receive benefits/entitlements			38	81%					
Medicare/Medicaid			31	67%					
On probation or parole			10	22%					
Use marijuana recreationally			40	85%					
Days of recreational cannabis use (past 90 days)			49.23 (0.00–90.00)	41.76					
Completers only (*N* = 40)			Baseline		3-Month Outcomes		Change		
			*M or n (range)*	*SD or %*	*M or n (range)*	*SD or %*	Test statistic**	*p*	Effect size**
Buprenorphine-positive UDS^t^			32	84%	31	82%	*X*^*2*^(1) = 0.20	0.65	
UDS-confirmed opioid use (excluding buprenorphine)			6	16%	2	5%	*X*^2^(1) = 4.00	0.13	
UDS-confirmed any drug use (excluding cannabis)			11	29%	9	24%	*X*^2^(1) = 0.50	0.48	OR = 1.67
UDS-confirmed cannabis use			29	76%	34	89%	*X*^*2*^(1) = 3.6	0.06	
Brief Pain Inventory – Short Form									
		Severity	5.18	2.09	4.39	2.28	*t*(39) = 3.44	< 0.01*	*d* = 0.54
		Interference	5.21	2.79	4.32	2.86	*t*(39) = 2.99	< 0.01*	*d* = 0.47
Pain Self-Efficacy Questionnaire			6.55	3.57	8.05	3.30	*t*(39) = −2.80	< 0.01*	*d* = 0.44
Brief Substance Craving Scale			2.15	2.88	1.78	2.95	*t*(39) = 0.69	0.49	*d* = 0.11
HRQoL (SF-36)									
	Physical functioning		59.25	24.38	70.25	25.47	*t*(39) = −3.39	< 0.01*	*d* = −0.54
	Role limitations—physical		36.87	41.60	50.00	45.64	*t*(39) = −2.63	< 0.05*	*d* = −0.42
	Role limitations—emotional		39.17	42.63	55.00	41.72	*t*(39) = −2.28	< 0.05*	*d* = −0.36
	Energy/fatigue		44.63	19.13	51.25	20.78	*t*(39) = −2.14	< 0.05*	*d* = −0.34
	Emotional wellbeing		57.90	19.28	67.50	19.95	*t*(39) = −3.24	< 0.01*	*d* = −0.51
	Social functioning		51.88	26.79	63.75	27.12	*t*(39) = −3.87	< 0.001*	*d* = −0.61
	Pain		39.94	26.27	57.25	28.89	*t*(39) = −5.01	< 0.0001*	*d* = −0.79
	General health		51.25	24.25	55.13	22.17	*t*(39) = −1.79	> 0.05	*d* = −0.28
Pittsburgh Sleep Quality Index – Global Score			12.38	4.40	10.95	4.95	*t*(39) = 2.08	< 0.05*	*d* = 0.33

^*^Significant at* P* < 0.05 level

^**^McNemar’s test or dependent samples t-test; Odds ratio or Cohen’s *d*

^t^n = 38 (two participants are missing UDS)

#### Changes in opioid use

*UDS-confirmed opioid use.* Rates of UDS-confirmed opioid use did not differ significantly from baseline to follow-up* X*^2^(1) = 4.00, *P* = 0.13 (exact test statistic). In total, 16% (*n* = 6) provided an opioid-positive UDS at baseline compared to 5% (*n* = 2) at Month 3. Among those with a status change from baseline to follow-up (*n* = 4), all individuals provided an opioid-positive UDS at baseline and an opioid-negative UDS at Month 3.

#### Changes in pain-related outcomes

*Brief Pain Inventory.* Pain severity scores decreased significantly from baseline (*M* = 5.18, *SD* = 2.09) to Month 3 (*M* = 4.39, *SD* = 2.28), *t*(39) = 3.44, *P* < *0.*01, Cohen’s *d* = 0.54. Similarly, pain interference scores decreased significantly from baseline (*M* = 5.21, *SD* = 2.79) to Month 3 (*M* = 4.32, *SD* = 2.86), *t*(39) = 2.99, *P* < *0.*01, Cohen’s *d* = 0.47.

*Pain Self-Efficacy Questionnaire.* Pain-related self-efficacy scores increased significantly from baseline (*M* = 6.55, *SD* = 3.57) to Month 3 (*M* = 8.05, *SD* = 3.30), *t*(39) = −2.80, *P* < *0.*01, Cohen’s *d* = 0.44.

#### Changes in other substance use-related outcomes

*UDS-confirmed any drug use excluding cannabis.* Rates of UDS-confirmed drug use excluding cannabis did not differ significantly at the two timepoints, *X*^2^(1) = 0.50, *P* = 0.48, *OR* = 1.67. A total of 29% (*n* = 11) provided a drug-positive UDS at baseline compared to 24% (*n* = 9) at Month 3. Among those with a *change* in status, five (13%) provided a drug-positive UDS at baseline and a drug-negative UDS at Month 3 and three (8%) provided the reverse.

*Secondary outcomes*. Craving scores were slightly higher at baseline (*M* = 2.15, *SD* = 2.88) than at the Month 3 follow-up (*M* = 1.78, *SD* = 2.95) but this difference was not statistically significant (*P* > 0.05, Table 1). Significant changes were observed in most domains of HRQoL and in global sleep quality from baseline to Month 3 (Table 1).

#### Product purchases

In total, 40 individuals (85%) made at least one MC purchase from baseline to Month 3. Three of the seven (43%) individuals who did not purchase MC products also did not complete the 3-month follow-up. Most participants (*n* = 31, 66%) purchased the discounted study medication, and flower product was purchased with the next highest frequency (*n* = 30, 64%).Those who purchased at least one product made 13.92 purchases on average (*SD* = 13.79; range = 1–64). The mean average THC content of purchased products was 23.05% (*SD* = 17.15; range = 0.33–65.70%) and the mean average CBD content was 0.64% (*SD* = 0.79; range = 0–4.00%).

## Discussion

As hypothesized, adults with OUD reported significant reductions in pain severity and interference and significant increases in pain self-efficacy over the 3-month period post-MC treatment initiation. Given that all measures of pain in this study improved following initiation of MC, as well as the potential for undertreated pain to precede or “trigger” opioid misuse, MC may represent an important tool for pain management in this population.

Overall, the urinalysis-confirmed rates of illicit opioid use were fairly low at both baseline and follow-up, and no participants initiated opioid use at Month 3; all participants providing a drug-positive UDS at Month 3 also provided a drug-positive UDS at baseline. MC was not associated with an increase in opioid or other substance use. Additionally, while drug craving scores did not change significantly, participants endorsed significant improvements in their sleep quality and quality of life. These mixed findings suggest MC use to potentially be associated with better sleep quality and diminished pain in OUD, two important factors that can contribute to illicit substance use. Craving levels, however, were also relatively low in the sample at baseline, which likely relates to their established use of buprenorphine. Greater changes in cravings and opioid use may be seen in a sample that more recently initiated buprenorphine for OUD.

The use of MC for OUD remains controversial (Wiese and Wilson-Poe 2018). The adjunctive use of MC alongside buprenorphine in patients with co-occurring OUD and pain represents one possible strategy to encourage better patient engagement and retention in OUD treatment. The stigma surrounding OUD and towards certain pharmacologic approaches to its treatment (e.g., methadone, buprenorphine) persist (McCurry et al. 2023; Stone et al. 2021; Madden et al. 2021; Burgess et al. 2021), which remain vastly underutilized despite their proven efficacy (Blanco and Volkow 2019). One barrier to MOUD use is the stigmatizing belief that opioid agonist therapy is akin to substituting one addictive substance for another, a belief that could similarly obstruct more widespread access and utilization of MC for OUD. However, adjunctive treatment approaches (Wiese and Wilson-Poe 2018) that have the potential to contribute to positive recovery-related outcomes are desperately needed to address this public health crisis. Concerns regarding both the safety and efficacy of this approach can only be properly addressed through the use of randomized, controlled clinical trials. Studies employing rigorous designs, with larger samples and for longer durations, are needed; however, the current Schedule I designation of products containing THC in the U.S. limits the use of randomized trials, and in turn, inhibits the collection of data critically required to comprehensively evaluate MC for OUD.

This study has several strengths. Foremost is that it is amongst the first to examine the potential additive effects of MC for pain and substance use in individuals receiving buprenorphine for OUD who also have chronic pain. This study also has several limitations. The observational design precluded comparisons to a control group and any attributions of causality. In addition, we provided participants the opportunity to purchase MC at a low cost, but the significant expense of MC physician evaluations and of MC products may be prohibitive for many individuals living with OUD and pain. Furthermore, participants were from one U.S. state; therefore, generalizability of study findings to individuals from other areas is unknown. Many participants reported recreational cannabis use at baseline. For these participants, it is possible that only the cannabis source(s), strain(s), route(s) of administration, guidance on type, frequency and dosing from a healthcare professional, and/or cost(s) may have changed between the two assessments. As such, it is possible that the clinical and psychosocial gains reported in this study could relate to changes in these factors. Finally, the sample size does not provide sufficient power to examine multivariate predictors of outcomes, which is important in determining if there are particular subgroups for whom MC may be more or less effective.

## Data Availability

The dataset is available at https://digitalcommons.pcom.edu/.
